# Enhancing mortality prediction after coronary artery bypass graft: a machine learning approach utilizing EuroScore

**DOI:** 10.2144/fsoa-2023-0152

**Published:** 2024-06-17

**Authors:** Emad Hijazi

**Affiliations:** 1 Department of General Surgery & Urology, Faculty of Medicine, Jordan University of Science & Technology, Princess Muna Al-Hussein Cardiac Center, King Abdullah University Hospital, Irbid, 22110, Jordan

**Keywords:** coronary artery bypass graft, EuroScore, machine learning, Middle-east, mortality

## Abstract

**Aim:** We developed a machine learning model using EuroScore assumptions and preoperative and intraoperative risk factors to predict mortality after coronary artery bypass graft (CABG). **Materials & methods:** We retrospectively examined data from 108 CABG patients at King Abdullah University Hospital, classifying them into risk groups via EuroScore and predicting mortality through random forest classification. **Results:** High-risk patients displayed longer surgical times and significant factors such as age and surgery choice. The median EuroScore was 0.95 (0.5–6.4). The model yielded high AUC scores (0.98, 0.95) indicating strong predictive accuracy. **Conclusion:** Our findings showed that the machine learning models combined with the EuroScore significantly improve post-CABG mortality prediction. For further validation, larger datasets are needed.

## Plain language summary

Coronary artery bypass grafting (CABG) is a well-established procedure for ischemic heart disease, yet with a 2.0% mortality risk within 30 days, its frequency has declined. EuroScore aids in predicting CABG mortality, considering various risk factors. Postoperative complications like myocardial infarction and heart failure can be severe, and aspirin use post-CABG may reduce mortality for up to 4 years, particularly in multivessel heart disease. This research project explores EuroScore's role in CABG mortality analysis, emphasizing its significance in assessing cardiac surgery quality. Prolonged aortic clamp and bypass times correlate with higher morbidity, while low-risk patients benefit from more left internal mammary artery grafts for improved survival.

## Abstract (plain language summary)

Coronary artery bypass grafting (CABG) is an established procedure to improve clinical outcomes in patients with ischemic heart disease.

EuroScore was developed to enhance patient selection for cardiac surgeries by predicting mortality risk after CABG, considering patient characteristics and risk factors.

CABG is considered a high-risk procedure with mortality rates of up to 2.0% within 30 days, leading to a decline in the number of CABG surgeries in recent years.

Postoperative complications following CABG can lead to serious morbidity and mortality, with myocardial infarction, heart failure, and atrial fibrillation being significant complications.

Aspirin use after CABG has been shown to lower mortality rates for up to 4 years, and patients with multivessel heart disease may benefit from CABG as the preferred treatment option.

The goal of the research project is to use EuroSCORE to analyze mortality risk following CABG and explore potential risk factors.

EuroScore is of prognostic significance after primary CABG, and its accuracy is critical for assessing the quality of care in cardiac surgery.

Prolonged aortic cross-clamp and cardiopulmonary bypass time are linked to higher morbidity and mortality rates after CABG.

The number of left internal mammary artery (LIMA) grafts is significantly higher in low-risk patients, providing a survival advantage.

Serious morbidity and mortality can result from postoperative complications following Coronary artery bypass grafting (CABG) surgery. With a mortality rate of up to 2.0% within 30 days, it is regarded as a high-risk procedure. Although over 200,000 CABG surgeries are still carried out in the USA every year, the number of CABGs has been steadily declining over the past 10 years [1,2].

CABG stands out as the most strongly supported method for enhancing outcomes of myocardial damage and neurocognitive deficit in individuals with ischemic heart disease [3]. It has been carried out, for more than 50 years and has improved considerably throughout that time [4]. The European System for Cardiac Operative Risk Evaluation (EuroScore) was created to enhance the patient selection process for cardiac surgeries as it predicts the mortality risk following the procedure [5]. Using the EuroScore, patient characteristics and risk factors related to cardiac and surgical procedures are gathered and weighted separately [6].

Myocardial infarction, heart failure, and atrial fibrillation were found to be the three distinct complications following CABG in a previous study [3]. According to current UK guidelines, patients who underwent CABG following a myocardial infarction should take aspirin for the rest of their lives as aspirin use after surgery has been proven to lower mortality rates for up to 4 years following CABG. Moreover, patients should also take another antiplatelet medication, such as clopidogrel, for up to 12 months [7]. When a patient has multivessel heart disease, CABG is the preferred course of treatment, and there is a relative extent of graft failure that raises mortality rates [7].

Consequently, intensive research is required to evaluate the patient characteristics and potential risk factors that predispose to mortality following the operation. The goal of our research project is to use the EuroScore model to carry out a descriptive statistical analysis to assess the mortality risk following CABG and to further explore the potential risk factors.

## Materials & methods

### Study design

This is a unicentric, retrospective study from medical records of the King Abdullah University Hospital (KAUH), the main hospital in north Jordan from January 2018 to December 2020. Exclusion criteria consisted of any patients with previous cardiac surgeries. The Institutional Review Board (IRB) at our hospital institute and university approved the study. The study was performed in accordance with the principles of the Declaration of Helsinki, and informed consent was waived by the IRB committee due to the retrospective nature of the study.

Demographic data were collected for age, sex, and body mass index (BMI), clinical data for the history of hypertension (HTN), diabetes mellitus (DM), smoking, previous myocardial infarction (MI), respiratory illness, history of cerebrovascular accidents (CVA), surgical variables including type of surgery, choice of surgery, time of anesthesia, surgery, aortic cross-clamp (ACC), cardiopulmonary bypass (CPB), and post-operative variables of complications, reopening and length of stay.

### Evaluation of mortality risk after CABG surgery

The European system for cardiac operative risk evaluation (EuroScore) is a risk model to calculate the mortality risk after cardiac surgery. The EuroScore database was developed based on the data of >14,000 patients. The risk factors used to develop the EuroScore model have been described elsewhere. Patients were considered with a low-risk of mortality if the EuroScore was <3, and medium-high risk if the EuroScore was >3.

### Statistical analysis

Continuous variables were described using mean ± standard deviation (SD) if the data were normally distributed according to the Shapiro-Wilk test, or median (Range) if the data deviated from normality. Categorical variables were described using frequencies (percentages). To analyze the correlation between demographic, clinical, and operative variables with mortality risk groups, Wilcoxon (Mann–Whitney *U*) test was used for continuous variables, chi-squared χ^2^, and the fisher-exact test was used for categorical variables if the category count was <5. A significant difference was considered with a p-value <0.05.

A machine learning random forest classification (RFC) ensemble was built to predict the mortality risk group (high-risk vs low-risk). The RFC model, hereafter referred to as RFC29, was trained on demographic, clinical, intra-, and post-operative variables including gender, age, history of DM, history of HTN, history of smoking, BMI, previous MI, respiratory illness, history of CVA, choice of surgery (urgent vs elective), type of surgery (CABG, CABG + valve, valve, or more than one valve), CPB time, aortic cross-clamp (ACC) time, anesthesia time, surgery time, pleural drain, inotrope support, complications, hyperthermia, time of mechanical ventilation (TOMV), site reopens, reintubation, length of intensive care unit stay (LOICUS), length of hospital stay (LOH), intra-aortic balloon pump (IABP), left ventricular (LV) impairment, internal mammary artery (IMA) harvest, and bleeding amount. Then, patients' data were randomly split into 80:20 training-testing sets. Model's performance was evaluated on the testing set using a mean bootstrap estimate with 95% CI, tenfold cross-validation (CV), classification report for precision and recall, and area under the receiver operating characteristics curve (AUC/ROC). The relative contribution of each feature was calculated using the permutation importance approach, by calculating the decrease in the model's score when shuffling the features. All machine learning analysis has been conducted using the scikit-learn package from Python version 3.10.8.

## Results

### Patients' characteristics

**Figure 1.**
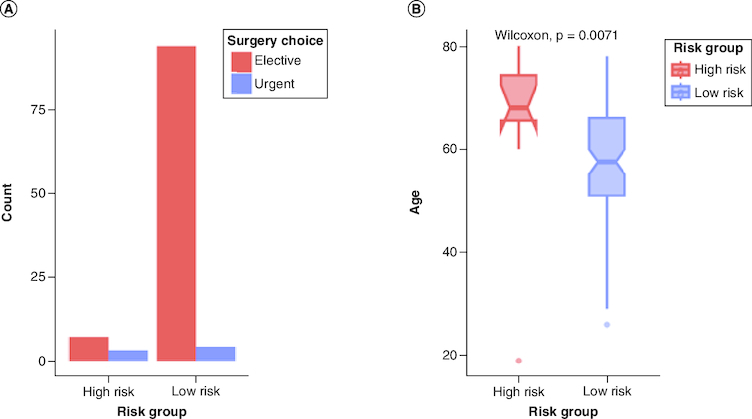
A comparison between high-risk and low-risk groups. **(A)** A bar plot for the frequency of surgery choice between risk group. **(B)** A box plot showing a significant difference in age between risk groups.

**Figure 2.**
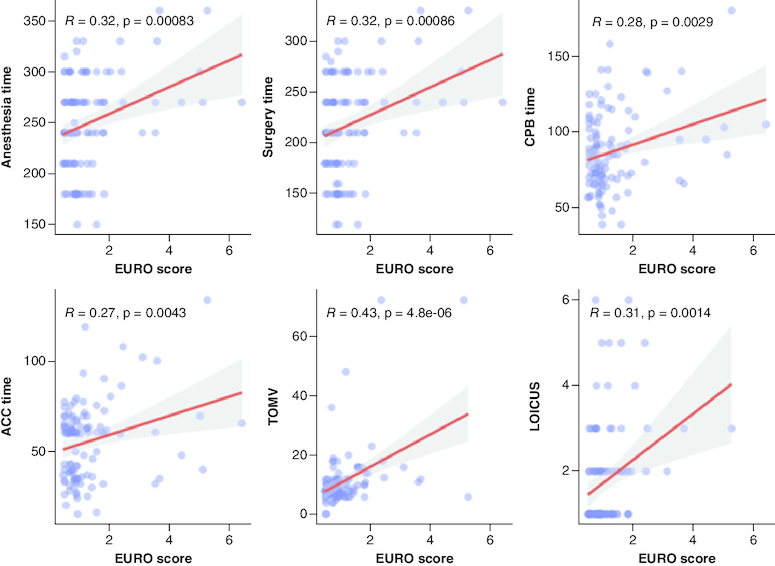
Scatter plots for the significant correlations between intra- and post-operative variables and the EuroScore.

A total of 108 post-CABG surgery were enrolled in this study. Of which, 92 (85.2%) were males and with a median (IQR) age of 58.5 (52–80). Eight (7.4%) patients had also valve surgery of which three patients with the aortic valve involved, five patients had mitral valve replacement. Four (3.7%) had more than one valve involved. Only seven (6.5%) of patients did an urgent CABG surgery. Five patients had both CABG and valve surgery, two of which had aortic valve replacement, and three patients had mitral valve replacement. Most patients had a history of HTN (73.1%) and DM (54.6%) while no patient was presented with a CVA. Based on the EuroScore, 98 (90.7%) patients were grouped as low-risk and ten (9.3%) patients as high-risk group. Only two patients in the high-risk group were females, with a significantly higher median age compared with the low-risk group (p < 0.01) as shown in Figure 1. None of the high-risk group had a history of respiratory illnesses, while 30 (30.6%) patients of the low-risk group presented with a history of respiratory disease as shown in Table 1. Anesthesia, ACC, surgery and CPB time showed a significant trend toward a positive correlation with the EURO score (p < 0.01, R >3) in addition to LOICUS and TOMV as shown in Figure 2.

**Table 1.** Demographic and clinical characteristics of patients included in the study.

Variable	All (N = 108)	Low-risk (n = 98)	High-risk (n = 10)	p-value
Gender (male), n (%)	92 (85.2%)	84 (85.7%)	8 (80%)	0.64
Age (year), median (range)	58.5 (52–80)	57.5 (26–78)	68 (19–80)	**0.007** ^†^
BMI, median (range)	28 (20–48)	28 (20–48)	26.3 (22–34)	0.22
Type of surgery, n (%)				
CABG	91 (84.3%)	84 (85.7%)	7 (70%)	0.12
CABG + valve	5 (4.6%)	3 (3.1%)	2 (20%)	
Valve	8 (7.4%)	7 (7.1%)	1 (10%)	
>1 valve	4 (3.7%)	4 (4.1%)	0 (0%)	
Surgery choice, n (%)				
Elective	101 (93.5%)	94 (95.9%)	7 (70%)	**0.02** ^†^
Urgent	7 (6.5%)	4 (4.1%)	3 (30%)	
History of smoking, n (%)	59 (54.6%)	56 (57.1%)	3 (30%)	0.18
HTN, n (%)	79 (73.1%)	71 (72.4%)	8 (80%)	1.00
DM, n (%)	59 (54.6%)	52 (53.1%)	7 (70%)	0.34
Previous MI, n (%)	28 (25.9%)	24 (24.5%)	4 (40%)	0.28
Respiratory illnesses, n (%)	30 (27.8%)	30 (30.6%)	0 (0%)	0.06
CVA, n (%)	0 (0%)	0 (0%)	0 (0%)	-
EuroScore, median (range)	0.95 (0.5–6.4)	0.92 (0.5–2.48)	4.06 (3.13–6.4)	**<0.001** ^†^

† Bold values indicate statistically significant p-values (p < 0.05).

CABG: Coronary artery bypass graft; CVA: Cerebrovascular accident; DM: Diabetes mellitus; HTN: Hypertension; MI: Myocardial infarction.

### Intra- & post-operative characteristics

**Figure 3.**
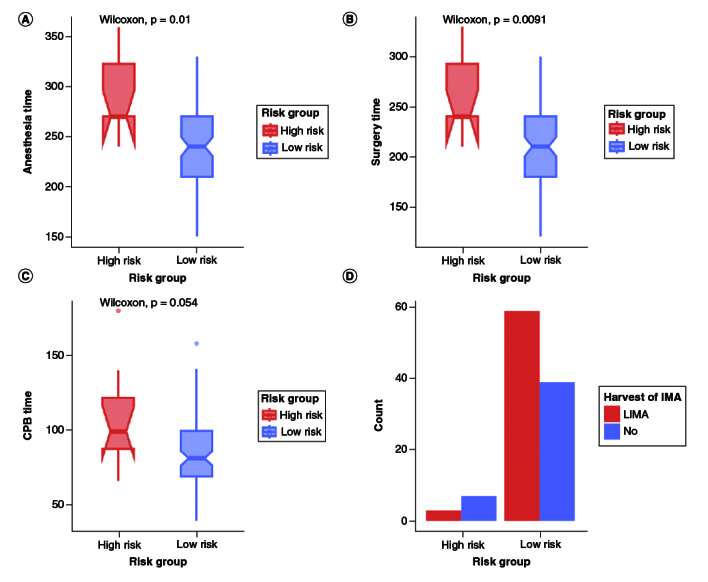
Boxplots for significant correlated intra-operative variables with risk groups. **(A)** High-risk group showed a significantly higher anesthesia time than low-risk group (p = 0.01). **(B)** High-risk group also showed a significantly higher surgery time (p < 0.01). **(C)** Partial significance in CPB time in which the high-risk group showed longer CPB time. **(D)** Bar graph showing the frequency of IMA harvesting between risk group where most of the low-risk group had LIMA harvest.

For the intra-operative variables, a total of 62 (57.4%) patients had left IMA harvest, out of which only three patients from the high-risk group underwent LIMA harvest, while 59 patients from the low-risk group underwent LIMA harvest (p < 0.001) as shown in Figure 3D. Only two patients had intra-aortic balloon pumps. High-risk group patients showed to be associated with an overall longer time for anesthesia, surgery, CPB and ACC, with a significantly higher anesthesia and surgery time compared with the low-risk group (median: 270 vs 240, p = 0.01; 240 vs 210, p < 0.01, respectively) and a partial significance in CPB time (median: 99 vs 81, p = 0.05) as shown in Figure 3A–C. Detailed intra-operative characteristics are shown in Table 2. For the post-operative characteristics, seven patients passed which were all from the high-risk group (70%), one patient from the low-risk group showed psychosis and one patient had a cerebrovascular accident. Most of the high-risk group (n = 9, 90%) had left ventricular (LV) impairment and were on inotrope support of dobutamine, dopamine and adrenaline. The median length of ICU stay (LOICUS) was significantly higher in the high-risk group (3 vs 1, p = 0.04) as shown in Table 3.

**Table 2.** Intra-operative variables between risk groups.

Variable	All (N = 108)	Low-risk (n = 98)	High-risk (n = 10)	P-value
Anesthesia time (min), median (range)	240 (150–360)	240 (150–330)	270 (240–360)	**0.01** ^†^
CABG Duration, median (range)	210 (120–330)	210 (120–300)	240 (210–330)	**0.009** ^†^
CPB time, median (range)	85 (39–180)	81 (39–158)	99 (66–180)	0.05
ACC time, median (range)	61 (15–135)	61 (15–120)	63.5 (32–135)	0.23
LIMA Harvest, n (%)	62 (57.4%)	59 (60.2%)	3 (30%)	**<0.001** ^†^
Bleeding amount (cc), median (range)	350 (0–1700)	350 (0–1700)	275 (0–775)	0.49
Pleural drain, n (%)				
One	73 (67.6%)	67 (68.4%)	6 (60%)	0.62
Two	32 (29.6%)	28 (28.6%)	4 (40%)	
Three	3 (2.8%)	3 (3%)	0 (0%)	
Hyperthermia (celsius), median (range)	33.2 (28–36)	33.2 (30–36)	33.4 (28–34.2)	0.65
**IABP, n (%)**	2 (1.6%)	1 (1%)	1 (10%)	0.18

† Bold values indicate statistically significant p-values (p < 0.05).

ACC: Aortic cross-clamp; CPB: Cardiopulmonary bypass; IABP: Intra-aortic balloon pump; LIMA: Left internal mammary artery.

**Table 3.** Post-operative characteristics.

Variable	All (N = 108)	Low-risk (n = 98)	High-risk (n = 10)	p-value
Reopening after CABG, n (%)	6 (5.6%)	4 (4.1%)	2 (20%)	0.10
Reintubation, n (%)	2 (1.9%)	1 (1%)	1 (10%)	0.18
LV impairment, n (%)	44 (40.7%)	35 (35.7%)	9 (90%)	**0.001** ^†^
Inotrope support, n (%)	40 (37%)	31 (31.6%)	9 (90%)	**<0.001** ^†^
Complications, n (%)	9 (8.3%)	2 (2%)	7 (70%)	**<0.001** ^†^
LOH (days), median (range)	5 (3–12)	5 (3–12)	5 (4–6)	0.94
LOICU stay (days), median (range)	1 (1–6)	1.0 (1–6)	3 (2–3)	**0.04** ^†^
Duration of mechanical ventilation (h), median (range)	9 (0.23–72)	9 (0.23–72)	12 (6–72)	0.16

† Bold values indicate statistically significant p-values (p < 0.05).

LOH: Length of hospital stay; LOICU: Length of intensive care unit stay; LV: Left ventricular.

### Predictive ML model of mortality risk

**Figure 4.**
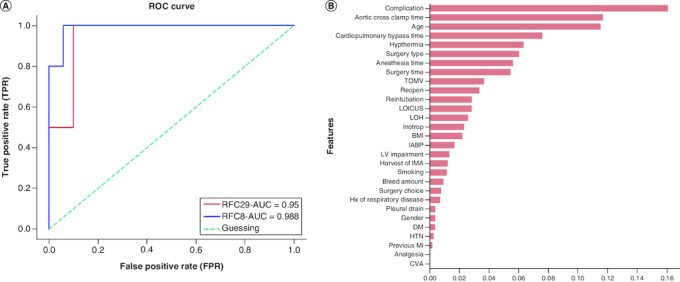
Machine learning models performance. **(A)** Area under the Receiver Operating Characteristic curve (AUC/ROC) for RFC29 and RFC8 models. The RFC8 model showed better performance and higher AUC. **(B)** Permutation importance based on the decrease in model's score when the feature is shuffled. Higher values indicate higher importance and contribution to model's prediction. Post-operative complications, aortic cross clamp time, age and cardiopulmonary bypass time showed higher contribution.

Two supervised ML ensembles were trained on 80% (n = 86) of the data and internally validated on 20% (n = 22). The RFC29 model which included 29 demographic, clinical, intra-, and post-operative features, predicted mortality risk with a high mean bootstrap estimate of 0.92 and 95% CI: [0.79–1.00], tenfold CV of 0.91 and an AUC of 0.95 as shown in Figure 4A. Permutation importance showed that post-operative complications, AAC time, age, CPB time, and hyperthermia were the highest contributing features as shown in Figure 4B. To test the predictive role of the top contributing features, the RFC8 model was trained on the top eight features with a mean decrease impurity higher than 0.06. The RFC8 model performed on the testing set with an inferior mean bootstrap estimate of 0.83 and 95% CI: [0.62–1.00], a superior AUC of 0.98, and a tenfold cross-validation of 0.96 as shown in Table 4.

**Table 4.** Evaluation metrics for machine learning models.

Ensemble	AUC	Bootstrap estimate (95% CI)	tenfold CV	Precision	Recall	F1-score
RFC29	0.95	0.92 (0.79–1.00)	0.91	0.91	1.00	0.95
RFC8	0.98	0.83 (0.62–1.00)	0.96	0.81	1.00	0.89

AUC: Area under curve; CV: Cross validation; RFC: Random forest classifier.

## Discussion

EuroScore is of prognostic significance after primary CABG. Its importance is limited to the lowest and the highest risk patients. Between 2011 and 2013, an analysis of the Society of Thoracic Surgeons (STS) Database revealed an operative mortality rate of 1.9% in isolated CABG patients. Compared with that, the operative mortality rate according to the New York Cardiac Surgery Reporting System in 2011 was 1.24% [8].

One of the biggest, most thorough and most accurate databases in European cardiac surgery history serves as the foundation for EuroScore, a prognostic system for evaluating heart surgery [9]. Operative or hospital mortality is widely recognized as an indication of the quality level of care in cardiac surgery [9,10]. It is critical that EuroScore be reliable because operative mortality must be related to the risk levels of the patients undergoing surgery [9]. Since there were no dead patients in the low-risk group, the EuroScore estimate of the high-risk group mortality was matched by the fact that seven out of the ten patients in the high-risk group who underwent CABG in our institution died. In a cohort study that was published in 2009, 30-day mortality was 0.8%, whereas EuroScore predicted that it would be 3.6% after CABG. Additionally, they noted that Norway's 30-day mortality rate following open heart surgery was lower than EuroScore prediction [10].

Prolonged aortic cross-clamp (ACC) and cardiopulmonary bypass time (CPBT) are linked to higher rates of morbidity and mortality, possibly as a result of the myocardial damage and inflammatory response brought on by cardioplegic cardiac arrest and cardiopulmonary bypass [11–13]. In our cohort, significant positive correlations between the EuroScore and anesthesia, ACC, surgery, and CPB time were observed. Patients in the high-risk group illustrated an overall longer time for ACC and CPB as well as longer anesthesia and surgery times when compared with patients in the low-risk group. However, only anesthesia and surgery times were statistically significant. Al-Sarraf *et al.* showed prolonged cross-clamp time significantly correlates with major postoperative morbidity and mortality in both low-and high-risk patients [14].

Our study showed that the number of LIMA harvests was significantly higher in the low-risk group. Which is a 15-year survival advantage was detected in patients who had received LIMA to the left anterior descending artery (LAD), and arterial grafting of the non-left anterior descending vessels compared with patients who had received venous grafting [15].

Post-operative left ventricular impairment, inotrope requirement, and length of stay in the ICU are higher in the high-risk group than in the low-risk group.

In 2012, the EuroScore I model underwent an update that resulted in the EuroScore II [16], which includes more precise and comprehensive patient, cardiac and operation-related factors, and a wider range of surgeries. It's been demonstrated to have higher predictive discrimination for intraoperative mortality [17]. Thus, a potential limitation of our study is that the data was gathered before the publication of EuroScore II. Other drawbacks of the study include its retrospective nature, which makes it susceptible to selection bias, and its small sample size. The generalizability of our findings may also be impacted by the fact that our study was restricted to a single institution and used a small sample size. The EuroScore II algorithm seems to possess greater complexity compared with its predecessors, despite having almost the same core set of risk factors. Some definitions have become more precise in this version. For instance, the symptomatic status now incorporates the NYHA class and the CCS Class 4, and the outdated category of unstable angina has been eliminated. Renal impairment is now classified considering creatinine clearance, and a more detailed categorization has been applied to define ejection fraction, pulmonary artery systolic pressure, and urgency [18,19].

The operation's mortality rate has a significant effect on society [10]. The benefit of our study is that it assesses the mortality rates for patients who underwent CABG at the leading tertiary hospital in northern Jordan. We implemented a machine learning model that uses algorithms to boost the prediction process on its own, making it simpler and more reliable [20]. Our project is the first machine learning project at our institution. We examined a variety of demographic, clinical, and intraoperative features when making our prediction, including ACC time, CPB time, hyperthermia, surgery type, and anesthesia type. Of these, ACC time, CPB time, and hyperthermia were particularly important as they influenced EuroScore.

Greater benefits for clinical management of CABG may arise from machine learning models used on larger datasets. Furthermore, to ensure a better prognosis following CABG, more research on the intraoperative and preoperative predictors of mortality should be conducted widely.

We validated the machine learning model internally; however, further external validation from larger datasets could help identify other factors associated with EuroScore prediction.

## Conclusion

EuroScore is a useful tool for determining the quality of CABG and is a reliable indicator of the mortality rate after the procedure. According to our study findings, the use of machine learning models could effectively result in significant improvements in the ability to predict the rate of mortality following CABG based on the intraoperative conditions that could be improved to obtain a better prognosis after the surgery.
